# ‘Dove Confident Me Indonesia: Single Session’: study protocol for a randomised controlled trial to evaluate a school-based body image intervention among Indonesian adolescents

**DOI:** 10.1186/s12889-021-11770-0

**Published:** 2021-11-16

**Authors:** Nadia Craddock, Kirsty M. Garbett, Sharon Haywood, Kholisah Nasution, Paul White, L. Ayu Saraswati, Chairunnisa Rizkiah, Bernie E. Medise, Phillippa C. Diedrichs

**Affiliations:** 1 Centre for Appearance Research, University of the West of England, Coldharbour Lane, Bristol, BS16 1QY UK; 2 Faculty of Medicine, Universitas Indonesia, Jakarta, Indonesia; 3 University of Hawaiʻi at Mānoa, Honolulu, USA; 4 Lazuardi Al Falah Depok Junior High School Indonesia, Kota Depok, Indonesia

**Keywords:** Body image, Indonesia, Southeast Asia, Low- and middle-income countries, Single-session intervention, School-based intervention, Adolescent mental health, Randomised controlled trial, Study protocol, Life skills education

## Abstract

**Background:**

Due to the prevalence and associated adverse health consequences of negative body image among adolescents globally, there is a need to develop acceptable, effective, and scalable interventions. School-based body image interventions delivered by trained teachers show promise in reducing negative body image in adolescents. However, there is currently a lack of evidenced-based body image interventions for use in low- and middle-income countries (LMICs). This paper outlines a protocol for the development and evaluation of *Dove Confident Me Indonesia: Single Session*, a single-session, teacher-led body image intervention for Indonesian adolescents.

**Method:**

The effectiveness of the intervention will be evaluated using a cluster randomised controlled trial design. Due to the COVID-19 pandemic, the trial will be conducted online. Trained teachers or school guidance counsellors will deliver the intervention. Self-report questionnaires will be collected at three time points: baseline, post-intervention, and two-month follow-up. The primary outcome is body esteem. Secondary outcomes are internalisation of appearance ideals, mood, engagement in life activities, tendency to engage in appearance comparisons, and skin shade satisfaction. A minimum of 1000 participants will provide 95% power to detect small-to-medium intervention effects. To account for attrition and potential internet issues, the sample will comprise of 2000 Indonesian adolescents in grades 7–9, attending state junior high schools in Surabaya, East Java. Quantitative and qualitative data on acceptability of the intervention will also be collected from teachers and students. Additionally, fidelity of lesson implementation will be assessed. This project received ethical approval from the Universitas Indonesia and the University of the West of England. The intervention will be disseminated in junior high schools throughout Indonesia via UNICEF’s Life Skills Education (LSE) programme, which will be freely available for teachers to download.

**Discussion:**

This paper presents *Dove Confident Me Indonesia: Single Session,* a culturally adapted school-based intervention designed to improve Indonesian adolescents’ body image. It details the plan for evaluation, highlighting the strengths and limitations of the proposed study design. It will be informative for others aiming to adapt evidence-based school curricula to promote well-being among adolescents in LMICs.

**Trial registration:**

NCT04665557. Registered 11th December 2020.

**Supplementary Information:**

The online version contains supplementary material available at 10.1186/s12889-021-11770-0.

## Background

Negative body image, often conceptualised as body dissatisfaction or low body esteem, is a prevalent concern among adolescents with far-reaching physical and mental health implications [1, 2]. Adolescents are particularly vulnerable to experiencing negative body image due to the physical, cognitive, emotional, and social changes characteristic of this developmental stage [3–5]. Studies indicate that as many as two-thirds of early- to mid-adolescents in high-income countries report negative body image [6–8]. This is concerning as longitudinal evidence indicates that negative body image during adolescence predicts poor outcomes including low self-esteem, depression, disordered eating, and the onset of risky health behaviours, such as smoking [7, 9, 10].

Negative body image among adolescents is not limited to those living in high-income and/or English-speaking countries. Al Sabbah and colleagues [11] found comparable rates of negative body image among adolescents in high-, middle-, and low-income countries. Further, data from cross-cultural studies show adolescents living in Asian countries including China, Japan, Korea, and Malaysia report similar or worse body dissatisfaction than those in regions such as Australia, Europe, the UK, or the US [12–15]. However, there is a current lack of adequately powered, theory-driven body image intervention research in low- and middle-income countries (LMICs) [16, 17]. This underscores the need for transparent intervention development and evaluation research focused on alleviating negative body image among adolescents in these settings.

### Indonesian context

Indonesia is an upper-middle-income, majority Muslim country in Southeast Asia and is the world’s fourth most populous nation [18]. Nascent studies indicate body image is an important health issue for Indonesian adolescents [19–22]. For instance, consistent with research on body image among adolescents in other Asian countries and in high-income, English-speaking countries, Azizah and Kristiutami [19] found 58.7% of Indonesian adolescents aged 14–19 (76.2% girls) in the city of Tangerang (a large urban centre in Greater Jakarta, Banten province) reported negative body image. Further, these studies present cross-sectional evidence that negative body image is correlated with disordered eating behaviours among Indonesian adolescent girls [21, 22] as well as low self-acceptance [20]. In addition, a recent U-Report poll^1^ [23] completed by over 3000 adolescents in Indonesia (53% girls) from all 34 provinces found 77% wanted to change something about their appearance. According to the same poll, just under half (47%) reported that worrying about how they look held them back from engaging in important life activities, and 89% expressed that they would like to learn ways to improve their body image.

Macro-level factors, including rapid urbanisation and modernisation, the globalisation of media and advertising, social media, and a legacy of colonisation have all been positioned as contributing to negative body image in LMICs, including Indonesia [24–27]. Consistent with global gendered appearance ideals, research indicates the thin ideal is dominant and often internalised by women in affluent, urban regions of Indonesia [28]. Meanwhile, Prianti notes that for men, the athletic body ideal is widely promoted in Indonesian media, in line with “westernized notions of masculinity” ([24], p116).

Beyond the influence of Eurocentric appearance standards, a Pan-Asian ideal, characterised by a blend of European and Asian features, is becoming dominant across Asia [29]. The Pan-Asian ideal places particular emphasis on the face and an “Asian White” skin shade ([29], p74). It can be attributed, in part, to the popularity of Japanese and Korean popular culture and a booming skin-lightening industry in Asian markets, including Indonesia [29, 30]. For instance, Korean pop culture (including K-pop and K-beauty) is recognised as an important influence on body image, particularly for younger generations in Indonesia and notably promotes a homogeneous appearance ideal emphasising an extremely thin body type and very light skin [31, 32]. Taken together, there is a clear need for evidence-based body image interventions in Indonesia to help young people resist unrealistic appearance ideals and bolster their overall body esteem.

### School-based body image interventions

Schools are an ideal environment to deliver universal, cost-effective body image interventions [16, 33]. Existing school-based interventions have found positive results in improving body image among adolescents in high-income, English-speaking countries, such as Australia and the UK (e.g., *Media Smart* [34]; *Me, You & Us* [35]; *Dove Confident Me: Single Session* [36] and *Dove Confident Me: Five Session* [37]). Further, evidence suggests that task-shifting programme delivery from highly trained subject experts (e.g., psychologists or body image researchers) to trained teachers and school staff is feasible and effective, thus promoting cost savings, increased reach, and sustainability [35–37]. Given the insufficent number of trained mental health providers coupled with the stigma associated with mental health conditions in LMICs including Indonesia [38, 39], universal, school-based interventions with teacher-led delivery stand to be a more acceptable and sustainable way to provide mental health-related education to adolescents [40].

### Single-session interventions

Single-session interventions are defined as the intentional delivery of a single-encounter intervention [41]. A growing body of evidence supports the capacity of single-session interventions to provide significant short-term improvements in youth psychopathology [41, 42], and more specifically, reduce negative body image among adolescents [16, 43]. On average, single-session body image/eating disorder prevention interventions are less effective than multi-session interventions [44, 45]. However, their value lies in their potential for greater accessibility and opportunities for dissemination at scale [41]. Particularly in LMICs, attention to the mental health of students is often not considered within the school curriculum [39, 46], thus bringing into question the feasibility and acceptability of running multi-session programmes. Single-session interventions may provide a more realistic intervention option for schools to provide mental health-related education among adolescents [40, 47]. Consequently, a single-session intervention stands to be a good first step in introducing body image curriculum in Indonesian schools.

### Dove Confident Me

In line with evidence highlighting the prevalence and adverse consequences of negative body image among young people in Indonesia and reports that Indonesian adolescents would like to learn ways to improve their body image, the development and evaluation of a body image intervention for adolescents in Indonesia is timely. Recognising this, a partnership has been initiated among body image and education experts, health professionals, UNICEF Indonesia, and the Dove Self-Esteem Project (DSEP; a social purpose industry initiative) to develop, evaluate, and disseminate a single-session body image intervention for delivery by schoolteachers in Indonesia. This intervention will be embedded within UNICEF Indonesia’s new Life Skills Education (LSE) curriculum, a government-approved programme for junior high schools, which will be disseminated to schoolteachers in Indonesia via online training in collaboration with the Indonesian government’s Ministry of Education and Culture. It will be freely available to all state junior high schools across Indonesia via a government website.

The *Dove Confident Me: Single Session* school-based intervention initially evaluated in the UK [36] served as the conceptual base for the development of the present single-session body image intervention in Indonesia, *Dove Confident Me Indonesia: Single Session*. In this first evaluation, Diedrichs and colleagues [36] found the intervention led to immediate improvements in British adolescents’ body esteem, mood, life engagement, as well as a reduction in disordered eating behaviours. Notably, results from the evaluation of * Dove Confident Me: Single Session* in the UK found more favourable outcomes when the teachers delivered the lesson compared to external researchers [36]. Moreover, the extended five-session version of *Dove Confident Me*, first evaluated in UK schools similarly yielding positive outcomes [37], has recently undergone cultural adaptation for use in urban Indian schools [48]. A recent randomised controlled trial (RCT) evaluating *Dove Confident Me India* [49] found both immediate and sustained improvements in body esteem, highlighting the potential for adapting *Dove Confident Me* across cultural contexts. In addition, findings from this research show that with cultural adaptation, *Dove Confident Me* can be an acceptable intervention in Asian contexts for both teachers and students [48], and therefore was chosen as the basis for the present intervention.

*Dove Confident Me Indonesia: Single Session* is a 90-min body image lesson designed to be delivered to mixed-gendered school classes of adolescents aged 12–15 years, facilitated by teachers or school guidance counsellors. *Dove Confident Me Indonesia: Single Session* integrates two evidence-based and theoretically grounded techniques to attenuate negative body image: media literacy and cognitive dissonance. Media literacy techniques include critical thinking and enhancing ‘realism scepticism’ to (1) increase recognition that digitally altered images are unrealistic and thus inappropriate targets for appearance comparisons and (2) draw attention to the underlying motives for advertisers to curate and edit images depicting appearance ideals in order to sell products [50]. Cognitive dissonance techniques aim to reduce individuals’ internalisation of societal appearance standards and subsequently improve body image by encouraging adolescents to actively challenge these ideals via verbal, written, and behavioural exercises [45]. Accordingly, *Dove Confident Me Indonesia: Single Session* has three learning objectives: (1) Identify the ways that media images can be altered to conform to and perpetuate unrealistic appearance ideals; (2) identify the negative consequences of unrealistic appearance ideals on themselves, their friends, and community; and (3) identify strategies to help themselves and others resist unrealistic appearance pressures.

### Paper and study aims

This paper aims to describe how *Dove Confident Me Indonesia: Single Session* was culturally adapted for use in Indonesian junior high state schools. It details the methods that will be employed to evaluate the intervention’s effectiveness and acceptability in a randomised controlled trial, as well as considerations for conducting this research during the COVID-19 pandemic. It also provides the plan for dissemination and highlights the strengths and limitations of the research trial study design.

The aim of the study outlined in this paper is to evaluate *Dove Confident Me Indonesia: Single Session*, as delivered online to students by schoolteachers or school guidance counsellors working in Indonesian state schools, in terms of intervention effectiveness and acceptability. Although originally designed to be delivered in a face-to-face format in school classrooms, project partners made a collaborative decision to evaluate the effectiveness of the intervention delivered in an online setting (i.e., via virtual video-conferencing classrooms) in response to the ongoing COVID-19 pandemic.^2^ Considering this adaptation, this study aims to assess the acceptability of the curriculum and pedagogy with students and teachers as well as the programme’s effectiveness in improving body esteem and related psychosocial outcomes. In line with previous evaluations of single-session body image interventions [36], we anticipate the intervention to provide short-term (up to two months follow-up) improvements in body image compared to a wait-list control group in order to assess the absolute impact of the intervention. Moreover, in line with the Tripartite Influence Model [51] and the targeted risk factors *Dove Confident Me Indonesia: Single-Session* aims to address, we anticipate a short-term reduction in internalisation of appearance ideals and appearance comparisons, alongside a reduction in the extent to which negative body image prevents adolescents from engaging in life activities relative to the wait-list control group. Finally, due to cultural adaptations of the original programme to challenge harmful skin shade ideals in Indonesia, we anticipate short-term improvements in skin shade satisfaction.

## Method

### Intervention design

To develop *Dove Confident Me Indonesia: Single Session* for use across Indonesian schools, it was necessary to culturally adapt the programme content, as well as modify the pedagogical format to accommodate modest school resources (e.g., no PowerPoint projector and limited internet access) so that the intervention is accessible to all schools across Indonesia, regardless of available resources. The main cultural adaptations are evident in the imagery and cultural examples used throughout the programme materials (i.e., photos and illustrations of Indonesians), role-play scenarios (e.g., aspirations to be a YouTuber), and discussions regarding cultural influences, such as K-pop. To accommodate limited resources, all video and PowerPoint content was removed, and more information, context, and imagery were added to the student worksheets.

Consistent with the development of other body image programmes including the original version of *Dove Confident Me: Single Session* [36], as well as general health intervention development best practice [52, 53], the adaption of *Dove Confident Me: Single Session* for an Indonesian context involved a rigorous and iterative community participatory design process. This included a careful review of existing effective body image interventions and the study of relevant theory, preliminary topic-related focus group discussions with Indonesian adolescents and teachers (led by UNICEF Indonesia and Girl Effect^3^ in partnership with Cimigo, a local research agency), as well as continuous consultation and collaboration among various academic, industry, and community stakeholders. Stakeholders in the present project include UNICEF Indonesia, which provides education, youth, health, and gender expertise; Indonesian academics, health professionals, and schoolteachers, who provide cultural context and expertise (authors CR, BEM, KN, and LAS); the Indonesian Ministry of Education and Culture; and Indonesian adolescents. Table 1 provides a summary of the intervention development process.

**Table 1:** Intervention Development Process Summary

Activity	By Whom	When
Preliminary focus groups with adolescents and schoolteachers in Sorong, West Papua and Bone, South Sulawesi exploring themes related to body image and appearance pressures and reviewing existing DSEP materials, including *Dove Confident Me*.	UNICEF Indonesia	March 2019
Literature review of theory and existing evidence-based body image interventions.	Authors: NC, KMG, SH	October–December 2019
Focus groups with adolescents and schoolteachers in Sorong, West Papua and Bone, South Sulawesi to test a selection of activities and to further understand the needs of Indonesian adolescents regarding body image as well as the needs of Indonesian teachers in terms of training requirements.	UNICEF Indonesia, Innovesia (research agency)	January–February 2020
Online focus groups with adolescent girls in Greater Jakarta exploring themes related to body image.	Girl Effect (non-profit organisation), Cimigo (research agency)	March 2020
Iterative content and language revisions of the curriculum based on the original evidence-based intervention working with both the English and Bahasa Indonesia versions of the programme.	Authors: NC, KMG, SH, PCD, CR, KN, BEM, and LAS	April–June 2020
Formal government review of curriculum involving ministry officials, education and health experts, and experienced teachers from different regions across the country.	Indonesia Ministry of Education and Culture	June 2020
Final content revisions to the Bahasa Indonesia language version of the curriculum plus final proofing (spelling and grammar).	Authors: NC, KMG, SH, PCD, CR, KN, BEM, LAS, UNICEF Indonesia, and translator (AW)	July 2020
Development of the design version of the curriculum (to include pictures and illustrations).	UNICEF Indonesia plus authors NC, SH, KMG, PCD, and DSEP for the Spot the Difference task	April–December 2020

Aligned with UNICEF Indonesia’s LSE curriculum, *Dove Confident Me Indonesia: Single Session* features skills-based, active learning via class discussions, small group activities, role plays, and individual tasks, all of which have embedded critical thinking and respect for diversity, tenets of the LSE curriculum. All materials (i.e., the teacher guide, teacher e-learning module, and student worksheets) are in Bahasa Indonesia, the national language of Indonesia. See Table 2 for an overview of the intervention content and associated techniques that address the targeted risk factors.

**Table 2:** Dove Confident Me Indonesia: Single Session Intervention Summary

Activity	Activity details	Therapeutic technique(s)	Risk factor target(s)
Introduction	1) Define body confidence (high body esteem). 2) Explain why body confidence is important. 3) Share information on the current state of body confidence among Indonesian boys and girls.	Psychoeducation	n/a
Activity One: Appearance Ideals – the ‘Perfect-Looking” Girl/Boy	1) In small same-gender groups, students generate and record physical attributes that comprise the 'ideal' appearance for girls or for boys in their student worksheets (see Fig. 3). 2) Guided class discussion critically exploring societal appearance ideals (i.e., changes over time, differences across cultures, relevant influences, are they realistic?).	Psychoeducation	Internalisation of appearance ideals
Activity Two: Is It Worth It?	1) Guided class discussion examining the costs of pursuing societal appearance ideals related to time, money, emotions, health, and relationships. 2) In small groups, students record the costs using silhouettes provided in their student worksheets (i.e., individual costs are recorded inside the silhouette; community and societal costs are recorded outside the silhouette). 3) As a class and then in pairs, students share why pursuing societal appearance ideals is a bad idea.	Cognitive dissonance	Internalisation of appearance ideals
Activity Three: Spot the Difference	1) In small groups, students analyse the differences in four pairs of photos (provided in their student worksheets) displayed in a 'before' and 'after' format in which the 'after' photo has been digitally altered (see Fig. 4). 2) Small groups discuss the techniques used on social media to change one’s appearance before and after a photo is taken; the impacts on young people; who benefits from digital alteration; and why it is unfair.	Media literacy Cognitive dissonance	Internalisation of appearance ideals Media pressure Comparison
Activity Four: Supporting Others to Be Body Confident	1) In pairs, engage in role plays of specific scenarios (provided in the student worksheets) in which one student must persuade the other to not compare themselves to media images and to not pursue societal appearance ideals. Each student must take on the role of the persuader. (See Fig. 5). 2) Guided class discussion on how media images are unreal and how comparison with them can be harmful.	Cognitive dissonance	Internalisation of appearance ideals Media pressure
Take-Home Challenge: Mirror Exercise	1) While standing in front of a mirror, students make a list of three things they value about their personality, three body parts they like because of what it allows them to do, and three body parts they like how they look. 2) While still in front a mirror, students say, “I like my XX” using the features they generated in their list.	Cognitive reframing (e.g., to think about body functionality) Self-affirmation	Self-esteem Attention bias
Conclusion	1) Each student writes down at least one thing they will do to promote body confidence (examples are provided). 2) Students sign and date these commitments. 3) Provide students an opportunity to share their commitments with the class.	n/a	n/a

In order to facilitate the lesson, all teachers and school guidance counsellors will be required to complete 4.5 h of online training, which consists of an online self-guided e-module (approximately 90 min) and two online synchronous training sessions (90 min each). The e-module training course in Bahasa Indonesia for the entire UNICEF Indonesia LSE programme including the body image lesson was designed by UNICEF Indonesia and Sentra Studia, a local digital agency. The study authors consulted on the design and content of the body image lesson section of this training. This complete training course will be available online by the Indonesian Ministry of Education and Culture for all state schoolteachers to complete prior to delivering the LSE curriculum in exchange for continued education credits. As part of this study, participating teachers and school guidance counsellors will be asked to complete the body image component of the e-module training only. Next, following the e-module training, participating teachers and school guidance counsellors will be required to attend two 90-min online group training sessions on the body image lesson, facilitated by a member of the research team (CR) on a virtual video-conferencing platform in Bahasa Indonesia. In the first session, teachers will learn about body image and will take part in a walk-through of the lesson (as ‘students’), which will be facilitated by CR. In the second session, teachers will practice delivering lesson activities, receive peer and trainer feedback on their teaching practice, and have the opportunity to ask any questions regarding the intervention.

### Internal pragmatic pilot study

Before commencing with the main cluster RCT evaluation, we will conduct a pragmatic internal pilot to assess the feasibility of recruiting and retaining participants to the online trial, as well as assessing preliminary acceptability of the intervention. The pilot will be conducted with two schools, with two classes per school (grades 7 and 8). The schools will be randomly allocated to either the intervention or wait-list control group. We expect approximately 60 students to participate per school, a sufficient sample size for testing the feasibility of a trial [54]. The pilot consent process will be the same as is detailed for the main trial. Two teachers or school guidance counsellors at the intervention condition pilot school will be trained to deliver the lesson by a member of the research team (CR). They will also be asked to complete the body image component of the UNICEF LSE teacher training e-module as previously detailed.

Participating students in the intervention condition school will take part in the *Dove Confident Me Indonesia: Single Session* lesson facilitated by one of the trained teachers with their class peers (up to 30 students per group) via a video-conferencing platform. They will complete the online survey assessment three times in line with the main trial timings: One week prior to the lesson (T1), 1 week after the lesson (T2), and 8 weeks after the lesson (T3). Lesson fidelity will be assessed by trained members of Cimigo, the local research agency conducting the research, following a checklist as well as a member of the research team (KN). Students in the intervention condition will also be asked to provide feedback on the acceptability of the lesson as well as the experience of participating in the lesson (including the use of breakout rooms) at the end of the T2 online survey. Further, two focus group discussions with students took part in the lesson (one group of girls, one group of boys) and two individual interviews with the teachers who delivered the lesson will be held to gather insights on the acceptability of the lesson. Participating students in the wait-list control condition will complete the three online survey assessments in parallel with the intervention condition (T1, T2, and T3). The school allocated to the wait-list control will be provided with lesson materials following T3. Feasibility feedback on the research procedure will be collected from Cimigo.

Progression criteria based on (i) recruitment, (ii) participant retention, (iii) adherence to intervention, and (iv) lesson fidelity will be used to ascertain how we proceed from the pilot to the main trial. In addition, though adverse effects of the intervention or research trial are not expected based on findings from similar previous intervention studies (e.g., [36, 37, 49]), the research team will screen the data for safety (e.g., trends indicating declining body esteem). Criteria will be rated for each parameter with a traffic light system from red (stop, major issue for the progress of the trial) through to amber (amend and proceed with caution) through to green (continue, no issues threatening the success of the trial) [55]. The progression criteria are presented in Table 3.

**Table 3:** Progression Criteria from Pragmatic Pilot to Main Trial

Criteria		Green	Amber	Red
Recruitment	How many parents consent and corresponding students assent (target *n* = 120)	**50% or above** Continue with main trial	**30 and 49%** Consult research team to advise on changes to recruitment plan	**Below 30%** The pilot study may need to be extended
Participant Retainment	Student completion up until T2 in both arms	**70% or above** Continue with main trial	**50–69%** Consult research team to advise on changes to survey administration protocol	**Below 50%** The main trial will need to reconsider how surveys are administered
Intervention Adherence	Number of students attending online lesson for the full duration (supplemented with qualitative feedback on online delivery of lesson)	**90% or above** Continue with main trial	**70–89%** Consult research team to advise on changes to lesson delivery	**Below 70%** The main trial will need to reconsider how lesson is delivered
Fidelity of Lesson Delivery	Percentage of the lesson covered by teacher, assessed by two fidelity checkers	**90% or above** Continue with main trial	**60–89%** Consult research team on possible changes to lesson intervention or training	**Below 60%** Fully reconsider lesson intervention/training

### Study design

The main study consists of a two-arm open parallel group cluster RCT with an intervention group and a lessons-as-usual wait-list control group. Stratifying based on school size (large, medium, small), randomisation of schools will be conducted by an independent researcher blinded to conditions through block randomisation, using random block sizes of two, four, and six. Randomisation of schools will be completed following confirmation that schools recruited to the trial meet eligibility criteria, and the school principal has provided informed consent.

**Fig. 1:**
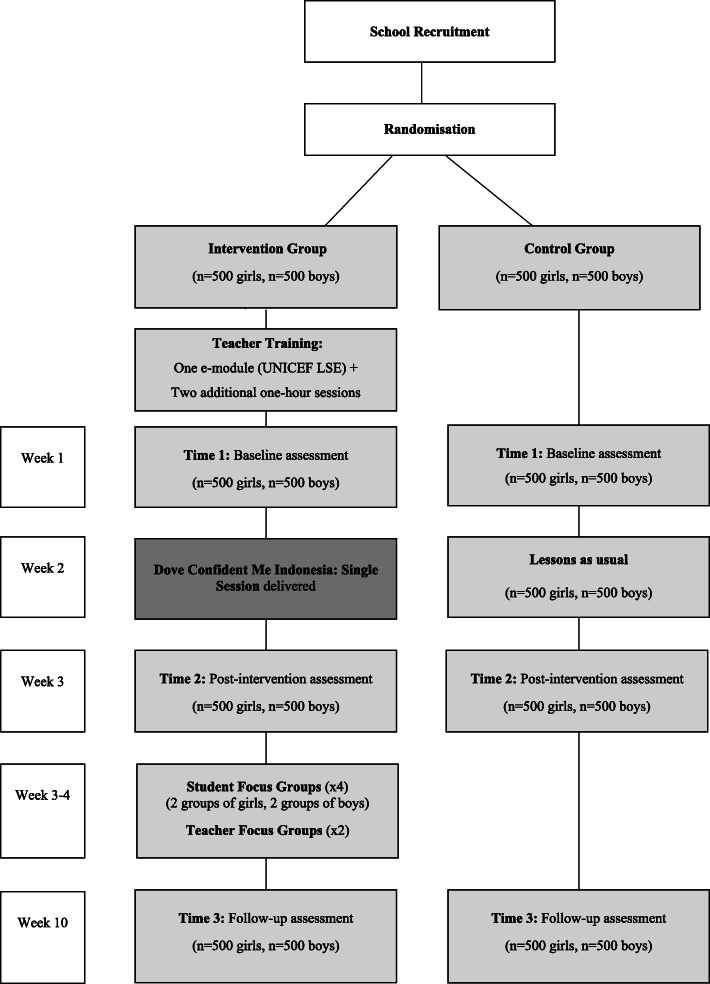
Study Design Flowchart

Cimigo and the study research team (i.e., study authors) will be informed of the study allocation, though will be blind to the size of each block during randomisation to minimise selection bias [56]. Participants will not be explicitly told their study condition, though will be made aware of the assessment time points and whether they receive the lesson between T1 and T2 (intervention) or after T3 (wait-list control). The statistician will be blinded to conditions during the trial and analysis. The trial will have assessments at baseline, post-intervention, and two-month post-intervention time points; see Fig. 1 for a flow diagram.

### Study setting and participant recruitment

This research will take place in Surabaya, Indonesia, a modern industrial city located on the northern shore of eastern Java. Surabaya is the second largest city in Indonesia and is home to approximately 2.9 million people in 2020 (85% majority Muslim, primarily Javanese). Notably, Java is Indonesia’s most populated island, home to approximately 56.1% of the population, and the Javanese people form the largest ethnic group in Indonesia [57].

Study participants will be 2000 Indonesian adolescents attending junior high schools in grades 7–9, alongside approximately 20–30 teachers and/or school guidance counsellors. Once permission to carry out this research has been obtained from the Surabaya District Education Office, junior high schools throughout Surabaya will be invited to participate based on the study eligibility criteria. School recruitment will be supported by UNICEF Indonesia. Eligibility criteria for schools and students can be seen in Table 4. Eligibility will be confirmed during the school recruitment process.

**Table 4:** Participation Eligibility Criteria at the School and Student Level

Inclusion Criteria	Exclusion Criteria
**School Eligibility Criteria**	
Co-educational state junior high schools (overseen by the Ministry of Education and Culture).	Madrasas (i.e., Islamic schools. Madrasas can be public or private. Public Madrasas are overseen by the Ministry of Religion). Private schools.
Have prior experience in delivering online instruction via video-conferencing platforms.^a^	Have delivered dedicated lessons on body image to current grade 7–9 students in the past year.
	Have taken part in UNICEF Indonesia’s LSE in the past year.
**Student Eligibility Criteria**	
Adequate literacy levels in Bahasa Indonesia (as determined by their teacher) to ensure students are able to independently read project materials.	^a^No access to an electronic device with an internet connection (e.g., tablet, laptop, desktop computer).
In grade 7–9.	

^a^criterion added due to COVID-19 pandemic restrictions

Teachers who are trained in delivering the body image lesson will identify students for the study using the eligibility criteria in Table 4. The teacher will send an electronic copy of the study information and consent letters to eligible students’ parent(s)/guardian(s). Active parental consent is a requirement from the Universitas Indonesia for young people under the age of 18. The information letter will detail the purpose and design of the trial, the risks and benefits to their child, how their child will be involved, how information will be stored, and ethical approval details. Parent(s)/guardian(s) will be given time to consider the information; the information sheet will provide contact details for members of the research team in Indonesia (authors BEM and KN) whom they can contact if they have any questions or queries at any point in the trial. Parent(s)/guardian(s) will be asked to confirm they have had the opportunity to read the information sheet provided and ask any questions. They will be asked to give their full name and consent for their child to take part in the research study. Parents are also asked to provide the full name of the child, so researchers can confirm consent with participant attendance at the teaching session and data collection time points. An example parent information and consent form is presented in Additional file 2. Informed parent consent will be provided electronically to the child’s teacher, who will pass this information to Cimigo. In addition, informed active assent will be electronically obtained from all students and matched with parent consent prior to data collection.

### Measures

Self-report measures to assess study outcomes were selected based on their use with adolescents in multiple studies internationally and are consistent with the *Dove Confident Me: Single Session* UK trial [37] to allow for comparison in outcomes.

However, to date, there are no existing validated measures of the outcome variables for use with adolescents in Bahasa Indonesia among Indonesian adolescents. Authors KMG, NC, SH, PCD, KN, BEM, and LAS are currently psychometrically validating the measures selected for the trial based on self-report data from Indonesian adolescents in Surabaya and Greater Jakarta, following a process outlined by Swami and Barron [58]. In brief, items from each measure were first analysed for cultural suitability by an expert Indonesian panel. Next, items were forward translated to Bahasa Indonesia by two independent translators fluent in both English and Bahasa Indonesia (their first language is Bahasa Indonesia) and back translated to English by a further two bilingual translators. Resulting translations from both rounds were reviewed by authors fluent in Bahasa Indonesia (LAS, KN, and CR), and any discrepancies in translations were subsequently resolved.

Next, acceptability interviews with 18 private and state school students aged 12–15 years were conducted by CR in Greater Jakarta, which provided support that the translated measures are likely to be understood and age-appropriate for Indonesian adolescents. Data has been collected from over 1000 Indonesian adolescents who completed the adapted and translated measures. Data is currently been prepared for analysis to assess the psychometric properties, reliability, and validity of each measure in this context, which will be completed prior to the analysis of the main trial. This research will be published in international peer review journals, with the findings informing how the measures will be utilised for purposes of the current trial.

#### Primary outcome measure: Body esteem

The primary study outcome will be body esteem, a specific component of body image, defined as a person’s self-evaluation of one’s body or appearance [59]. Body esteem will be measured using a culturally adapted Bahasa Indonesia version of the Body Esteem Scale for Adolescents and Adults (BESAA) [59]. Specific items and subscales to be used during the analyses will depend on the findings of the psychometric testing of the measure, as previously described. The self-report items are rated on a five-point Likert-type scale from 1 (never) to 5 (always). Negatively worded items are reverse scored and higher mean scores indicate greater body esteem.

#### Secondary outcome measures

Mood will be assessed using a culturally adapted Bahasa Indonesia version of the Positive and Negative Affect Schedule for Children (PANAS-C) [60]. Participants will rate one-word descriptors of positive affect (e.g., “cheerful”) and negative affect (e.g., “miserable”) on a five-point Likert-type scale from 1 (never) to 5 (always). Scores of positive items and negative items will be averaged as separate subscales so that higher mean scores of the positive items indicate greater positive affect and higher mean scores of the negative items indicate greater negative affect. As described, the specific number of items that will make up each subscale is currently unknown.

Internalisation of societal appearance ideals will be assessed using a culturally adapted Bahasa Indonesia version of the internalisation-general subscale of the Sociocultural Attitudes Towards Appearance Questionnaire-3 (SATAQ-3) [61]. Participants will be asked to indicate agreement with items (e.g., “I wish I looked like the models in music videos”) on a five-point Likert-type scale from 1 (totally disagree) to 5 (totally agree). Higher mean scores indicate greater internalisation of societal appearance ideals.

Reduced life engagement related to negative body image will be measured using a culturally adapted Bahasa Indonesia version of the Body Image Life Disengagement Questionnaire (BILD-Q) [62]. Participants will be asked to indicate the frequency with which they have not participated in an activity (e.g., giving an opinion, going to the doctor) due to worrying about their appearance by using a four-point Likert-type scale from 1 (not at all) to 4 (all the time), with higher mean scores showing poorer life engagement due to negative body image.

A purpose-built skin shade chart will be used to assess change in skin shade satisfaction. The chart will comprise of nine skin colours (using colours from The Pantone Skin Tone Guide: www.pantone.com/) from dark (1) to light (9).^4^ Participants will be asked to indicate which skin shade best represents their “current skin shade” and which best represents the skin shade they would like to have – their “ideal skin shade”. This will allow for a current ideal skin shade discrepancy score to be calculated as an indicator for skin shade satisfaction. As this measure has not yet been utilised among Indonesian adolescents, two further items will be included to assess the validity of this measure. The first additional item (i.e., “How dissatisfied or satisfied are you with the colour of your skin?”) provides response options on a five-point Likert-type scale 1 (very dissatisfied) to 5 (very satisfied). The second item (i.e., “Which of the following statements do you agree with the most?”) offers the following response options on a three-point Likert-type scale: “I would like my skin colour to be lighter”; “I would like my skin colour to stay the same”; and “I would like my skin colour to be darker”. These items will be corroborated with the current ideal discrepancy scores; provided there is a good correlation among scores, the current ideal discrepancy score will be used as the outcome variable on skin shade.

Finally, two purpose-built items will be included to assess the change in participants’ tendency to engage in appearance comparisons in the past week (i.e., “I compare my appearance to celebrities and influencers” and “I compare my appearance to people my age”). The two items will be rated on a five-point Likert-type scale 1 (never) to 5 (always). Provided there is good internal reliability between the two items [63], scores will be averaged, with higher mean scores indicating a greater tendency to engage in appearance comparisons.

#### Demographic information

At baseline (T1), participants will be asked to self-report demographic information (i.e., age, gender, ethnicity, and religion), in addition to their height and weight (if known) to calculate their body mass index (BMI).

### Intervention acceptability

Quantitative and qualitative intervention acceptability data will be collected from students and teachers in the intervention condition. Acceptability questions, adapted from the *Dove Confident Me: Single Session* UK trial [36] will be incorporated into the post-intervention (T2) assessment survey. First, students will be asked to rate the lesson using a five-point Likert-type scale from 1 (strongly disagree) to 5 (strongly agree) on perceived enjoyment, helpfulness, understanding, comfort, teacher competence, topic importance, the likelihood to take future action to improve body confidence in themselves or others, and whether they will recommend the lesson to a friend. Second, students will be invited to provide free-text responses on what they liked most and least about the lesson, as well as their preference regarding the number of body image lessons using a three-point Likert-type scale (i.e., “I would like more sessions on body image”; “I think one session on body image is enough”; and “I think we do not need any sessions on body image”). Third, they will be asked what they perceived as the key messages of the programme via a free-text response to assess whether the learning objectives were understood. Fourth, students will be asked to rate the acceptability of the online delivery format of the lesson on a three-point Likert-type scale (i.e., “I had a good connection”, “My connection was okay”, and “I had a poor connection”). Finally, a free-text option will also be provided to describe any connectivity issues they might have experienced.

Teachers delivering the lesson will be asked to complete a short acceptability questionnaire online to capture their perspective of the programme in terms of the relevance of the topic for their students, adherence to the content, student engagement, and perceived student understanding. Then, in-depth acceptability data will be obtained from a subset of students (approximately *n* = 16) in a series of single-gender online focus groups (2 x focus groups with girls; 2 x focus groups with boys) led by a trained researcher. Similarly, a subset of teachers (approximately *n* = 6) will participate in an online acceptability focus group. Schedules for both the student and teacher focus groups are presented in Additional file 3: Acceptability Focus Group Schedules.

### Intervention fidelity

The extent to which facilitators deliver the intervention as intended (i.e., intervention fidelity) will be assessed based on a refined protocol from previous *Dove Confident Me* trials [36, 37]. Approximately half of the lessons, which will include various lesson facilitators and a range of student grades, will be assessed in real time by a trained fidelity assessor (a member of the Cimigo team) ensuring each lesson facilitator (i.e., teacher or guidance counsellor) is assessed at least once. In addition, approximately 50% of lessons will be video recorded (25% of lessons assessed in real time and 25% of lessons not assessed) to allow for additional checks by a second trained fidelity assessor (either a member of the research team fluent in Bahasa Indonesia or a member of Cimigo) for the purposes of establishing inter-rater reliability via intra-class correlations (ICC) for 25% of the lessons. These will be interpreted based on established criteria: ICC values less than 0.5 to indicate poor reliability; values between 0.5 and 0.75 to indicate moderate reliability; values between 0.75 and 0.9 to indicate good reliability; and values greater than 0.9 to indicate excellent reliability [64].

A standardised checklist prepared by study researchers will be followed, whereby fidelity assessors will check off the tasks and activities as outlined in the teacher guide as they happen during the lesson. Fidelity assessors will also note how long each of the six lesson sections takes the teacher to deliver. Furthermore, fidelity assessors will assess 12 teacher characteristics deemed important for effective delivery using five-point Likert-type scales from 1 (not at all) to 5 (very much). Example characteristics include “ideas expressed clearly” and “demonstrated enthusiasm for the material”. Fidelity assessors will rate on a 10-point Likert-type scale the extent they think the learning outcomes were achieved from 1 (not at all) to 10 (very much so). Finally, fidelity assessors will report if there were any deviations from the teacher guide or technological difficulties during the lessons.

### Procedure

Following randomisation, teachers and/or school guidance counsellors at the intervention-assigned schools delivering the intervention will be provided with access to UNICEF’s LSE e-module training curriculum to work through the body image lesson content at their own pace, as well as two 90-min live online training sessions (provided by author CR) via a video-conferencing platform. Teachers in the lessons-as-usual wait-list control group-assigned schools will be offered the training following the completion of the trial.

After parental/guardian consent is obtained and student assent is provided, students participating in the trial will complete an online survey on their own electronic device via Qualtrics software at three time points: T1 (pre-intervention), T2 (post-intervention), and T3 (two-month follow-up). Each survey will comprise the measures previously detailed in addition to some random attention-check items, with each survey expected to take approximately 30 min to complete. Survey data collection will be online in class groups led by a researcher via a video-conferencing platform. Only the lead researcher and teacher will be requested to have their videos on for this task. Students can choose whether they want their videos on or off. The researcher will verbally confirm student assent before explaining the format of the survey; the class teacher will also be online to support the process. Students will be expected to stay online while they complete the survey in a separate browser window on their device, and the researcher will be available to answer any questions via a private text function. Teachers will ensure only students with parent/guardian consent are present at each data collection time point in both conditions.

At intervention-allocated schools, participating students will take part in *Dove Confident Me Indonesia: Single Session* in groups of 20–30 students 1 week after completing the T1 online survey assessment. The lesson will take place via a virtual live class setting using video-conferencing software during school time and teachers will be responsible for ensuring only students with parent/guardian consent attend. Teachers may deliver the lesson to multiple groups of students at their school. Fidelity assessors will be present at approximately half of the lessons. Teachers will be requested to have their videos on during the lesson to enhance engagement. Bandwidth allowing, students will be invited to have their video on to simulate an in-person classroom environment, though they will be shown how to turn off their self-display if they would prefer to not see themselves throughout the lesson. Prior to the lesson, all students will be sent paper copies of the student worksheets via courier. Student survey acceptability feedback will be completed online at the end of their T2 assessment. Teacher acceptability feedback via an online survey will be completed after delivering the lesson on the same day or at a maximum of up to 1 week later. Student acceptability online focus groups will take place within a week following the T2 survey. Teacher acceptability online focus groups may take place before or after the T2 survey depending on scheduling.

At wait-list control-allocated schools, participants will complete the three assessments (T1, T2, and T3). After T3, teachers will be offered the lesson training and students will be provided with the lesson materials. Teachers can decide a suitable time to schedule the lesson.

**Fig. 2:**
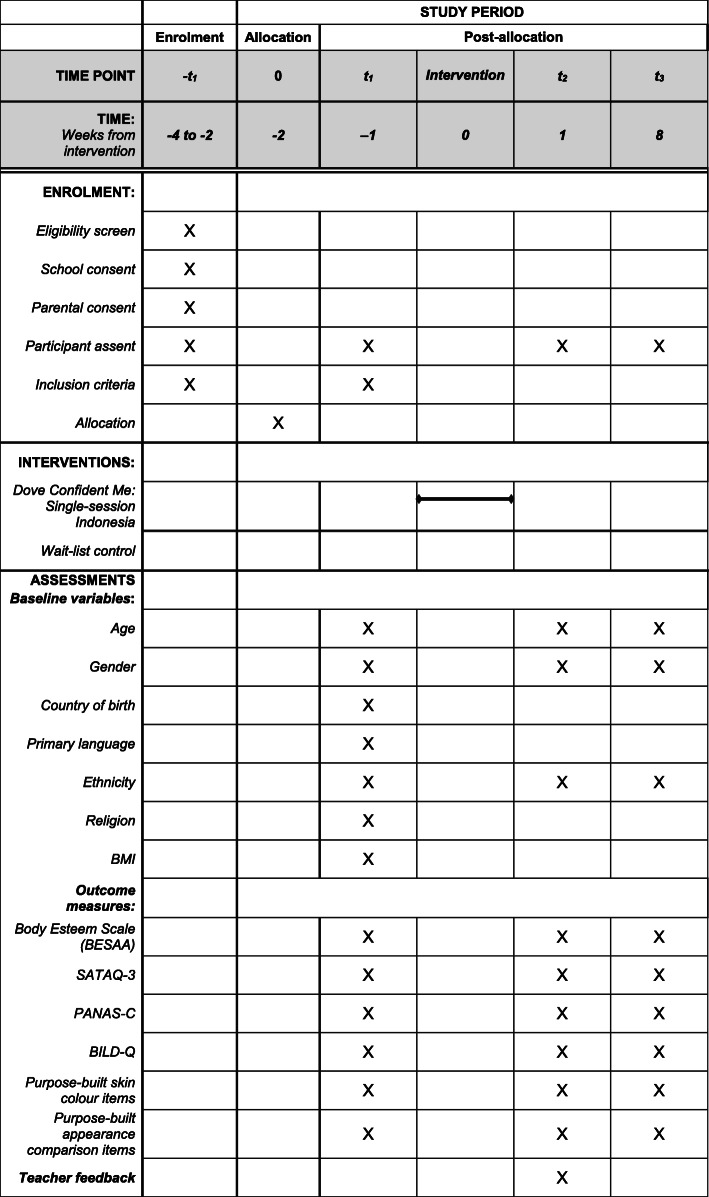
Schedule of Enrolment, Interventions, and Assessments

All adolescents involved in the trial will be provided an electronic copy of a debrief sheet, which includes sources of support (Additional file 4: Debrief Sheet) and will be informed to contact their teacher or school guidance counsellor should they have any concerns in line with usual school procedures. All participating adolescents, teachers, and schools will receive a certificate on behalf of all research partners for their contribution to this research upon the completion of the trial. See the schedule of enrolment, interventions, and assessments in Fig. 2. The recommended Standard Protocol Items: Recommendations for Interventional Trials (SPIRIT) 2013 checklist with items to address in clinical trial protocols is provided as Additional file 1.

**Fig. 3:**
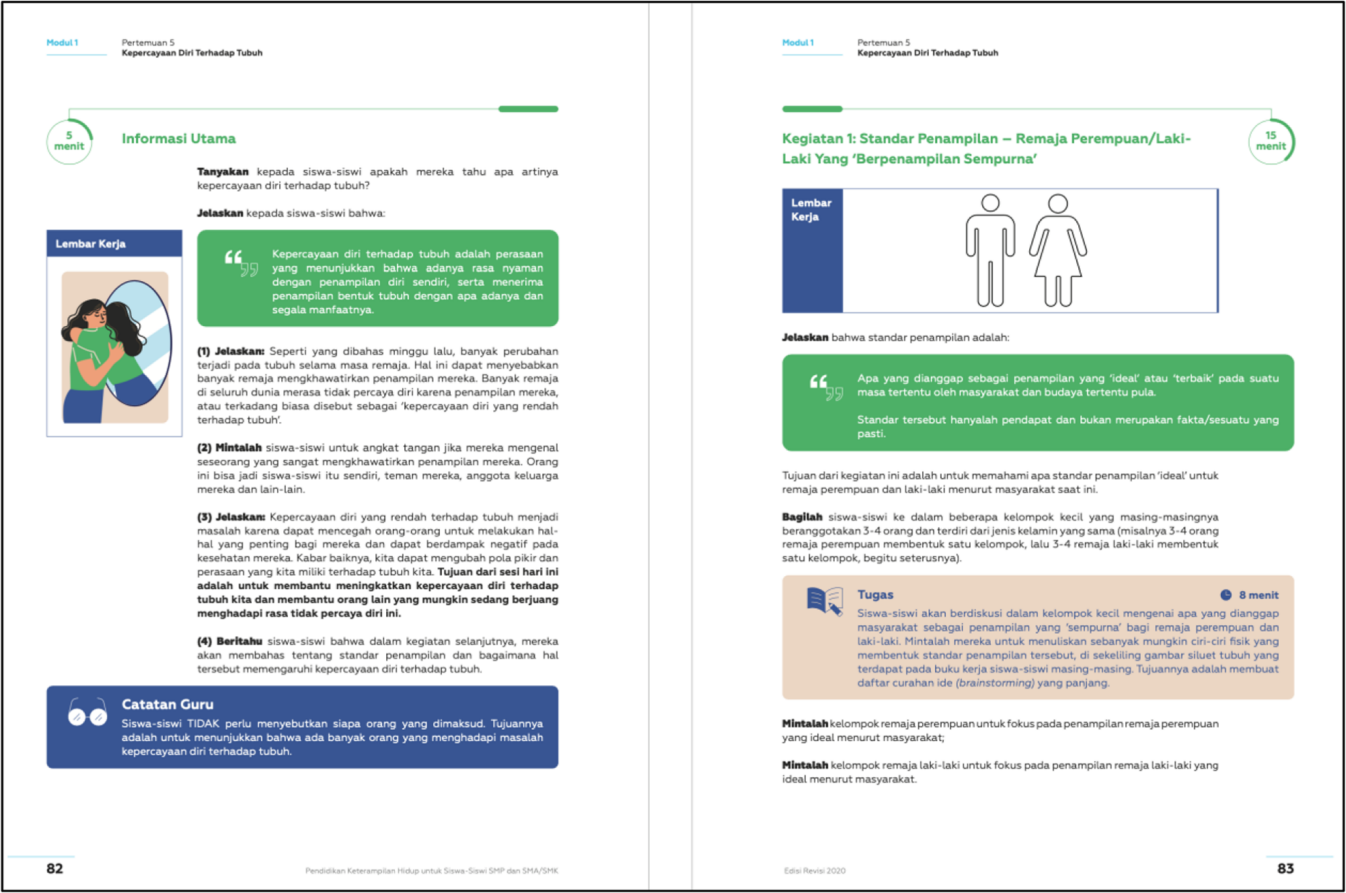
An Excerpt of the Teacher Guide Featuring the Introduction and Part of Activity One

**Fig. 4:**
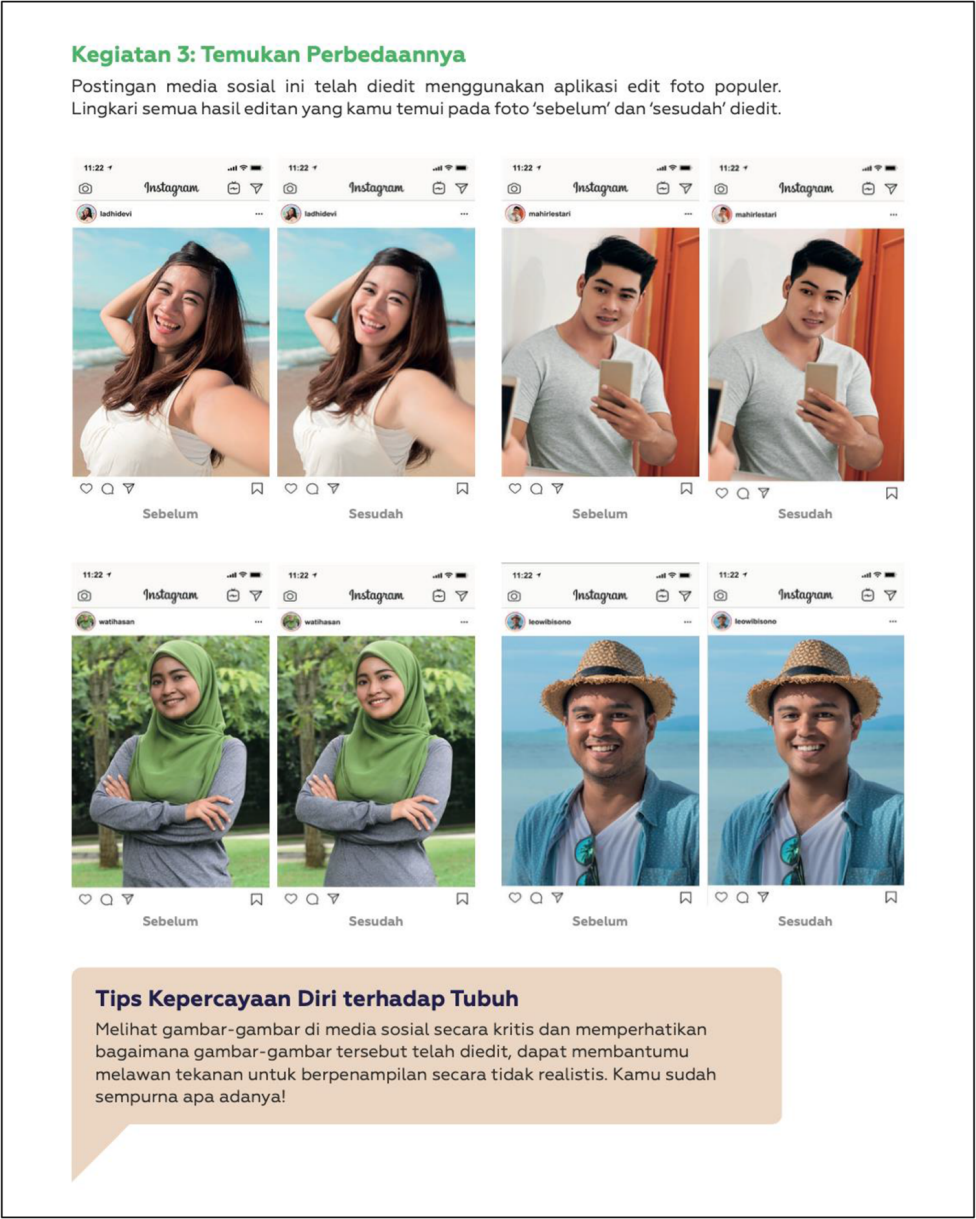
The Before-and-After Social Media Photos Provided for Students in Activity Three

**Fig. 5:**
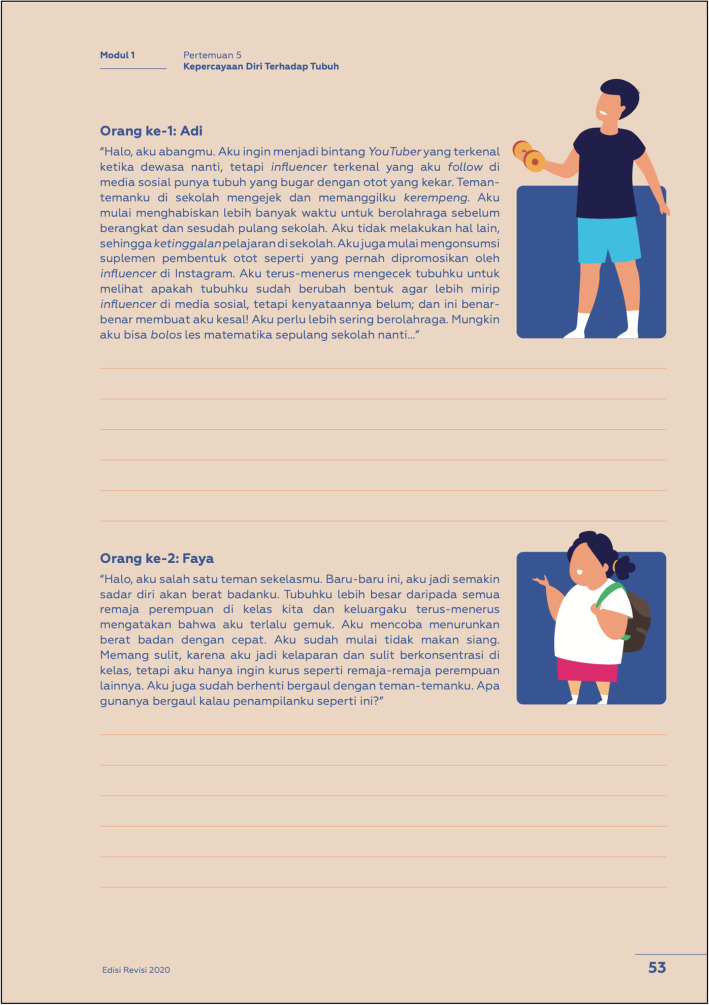
The First Two Role-Play Scenarios Provided for Students in Activity Four

### Ethics and permissions

This trial received ethical approval from the Universitas Indonesia (KET-1373/ UN2.F1/ETIK/PPM.00.02/ 2020) and the University of the West of England (HAS.20.05.174). Approval for field data collection in junior high state schools in Surabaya was attained from the Surabaya District Education Office. The approval process from the Surabaya District Education Office involved obtaining a research permit from Bakesbang (National Unity and Politics Office) Surabaya and Bakesbang East Java. The application for this permit was led by authors at the Universitas Indonesia (BEM and KN), supported by UNICEF, Cimigo, and the UK research team. This project is registered as a clinical trial via clinicaltrials.gov: NCT04665557.

### Data monitoring and management

Based on previous evaluations of iterations of *Dove Confident Me* [36, 37], the study procedure and intervention are considered to be low risk for participant safety. Therefore, it was decided that an independent data monitoring committee is not required. However, the research team will monitor the data quality for completeness, consistency, and plausibility throughout the data collection period.

Electronic consent data will be kept securely by Cimigo for a five-year period in line with the Universitas Indonesia’s ethical requirements; it will not be shared among the research team. All survey data will be securely entered online using University of the West of England-approved Qualtrics software. Unique codes will be created by adolescents in order to match their responses across time points. Teachers will be allocated unique codes based on their school and class to allow researchers to match teacher responses to their classes’ data. Personal data (e.g., names, WhatsApp numbers, email addresses) of participating teachers will be held by Cimigo only and will be deleted upon the completion of the trial. Personal data (e.g., linking names with WhatsApp numbers and email addresses) of participating students to coordinate attendance at the lesson and data collection time points will be kept by teachers only.

Survey data from participants will be stored directly on password-protected Qualtrics accounts accessible only to the University of the West of England research team. Once downloaded, data will be stored on university-approved secure cloud storage (i.e., OneDrive). As personal data will not be requested from adolescents, the survey data should be anonymous. However, the data will be screened for personal data collected inadvertently (e.g., in qualitative free-text response boxes). Any personal data will be removed from the dataset at the earliest possible stage. Once screened, data will be securely shared with project partners at the Universitas Indonesia via OneDrive.

Video recordings of the lesson used to complete fidelity assessments will be taken by Cimigo and will not be shared. Once the fidelity intra-rater reliability assessments are complete, these videos will be permanently deleted. Similarly, audio recordings from the acceptability focus groups taken by Cimigo will not be shared with the wider research team. Audio data will be transcribed in Bahasa Indonesia by Cimigo and any personal data (e.g., participant names) will be removed. The anonymised transcripts will then be shared with research team members fluent in Bahasa Indonesia (LAS, KN, and CR). Researchers will review the transcripts within a month of receipt and check any points of clarification with Cimigo, who will review the audio recordings. Once all transcripts have been approved, the audio recordings will be permanently deleted.

### Sample size calculation

A meta-analysis of 50 RCTs evaluating single-session adolescent psychological health interventions [41] reported a mean effect size of Hedges *g* = 0.32, which can be interpreted as a small-to-medium effect size (small effect size = 0.2; medium effect size = 0.5). This is consistent with findings from the RCT evaluation of *Dove Confident Me: Single Session* [36], which reported standardised effects ranging from small to small-to-medium for outcome measures. Based on a more conservative, lower bound of d = 0.2, a sample size of *n* = 500 girls and *n* = 500 boys per arm would provide in excess of 95% power over any positive range of correlation between baseline and outcome data at T2 and would have in excess of 90% power for separate analyses for girls and boys. We will oversample to mitigate the effects of loss to follow-up due to student absence or technical difficulties to increase population coverage and external validity. Therefore, the target study sample size will be approximately 2000 adolescents: 1000 (500 girls, 500 boys) in the control condition, 1000 (500 girls, 500 boys) in the intervention condition. It is estimated that 20 eligible schools will be recruited to achieve this sample size.

### Data analysis

Data preparation and assessment of baseline equivalence will be undertaken using SPSS 28.0 (IBM Corp, 2021). Data will be screened to ensure coding fidelity and data veracity. Data validity checks will be undertaken, and derived outcome scale data will be examined for the presence of outliers and any unduly inferential observations and to assess the distribution of scores. Analysis will proceed on an intention-to-treat basis. Analysis will be conducted with and without clusters as random effects to determine any significant impact [65]. Missing data will be assessed across outcomes and time points. A sensitivity analysis to missing data will be performed. Attention check data (greater than 70% successful completion of attention checks) will form a per-protocol analysis.

The primary analysis will be a linear mixed model with random intercepts. The model will include the three main effects, three two-way interactions, and three-way interaction for randomised arm (between-subjects fixed effect factor with two levels intervention and control), study phase (repeated measures fixed effect factor with two levels T2 and T3), gender (between-subjects fixed effects factor with two levels, male and female), including a baseline measure as a covariate, and the three two-way interactions between covariate and each of randomised arm, gender, and phase. A priori analyses will proceed primarily comparing the two randomised arms at T2 controlling for baseline covariate (a) ignoring gender (b) split by gender and (c) incorporating gender as a factor. These analyses will be repeated at T3. These prior reasoned analyses will use ANCOVA. The ANCOVA model will be extended to include a baseline by randomised arm interaction term if the homogeneity of regression lines assumption is not tenable. All models will be screened for gross deviations from underpinning assumptions to ensure valid statistical inferences are drawn.

### Dissemination

Findings of the study will be presented at international conferences and published in peer-reviewed journals. In addition, final reports will be written for the Indonesian Ministry of Education and Culture as well as the Surabaya District Education Office. Further, in the interest of knowledge exchange, results and learnings from this research will be shared on freely available podcasts and blogposts. Lesson material will be freely available for Indonesian state schoolteachers when they register for the UNICEF Life Skills Education programme via an Indonesian Ministry of Education and Culture webpage.

## Discussion

The aim of this protocol paper is to present the *Dove Confident Me Indonesia: Single Session* intervention, detailing how it was developed, how it will be evaluated in terms of effectiveness and acceptability among Indonesian school students and teachers, and how it will be disseminated. *Dove Confident Me Indonesia: Single Session* aims to provide a culturally adapted intervention delivered by teachers to improve Indonesian adolescents’ body image and related psychosocial outcomes. To the best of our knowledge, this cluster RCT will be the first rigorous effectiveness and acceptability evaluation of a school-based body image lesson in Indonesia. Learnings from this study will be applicable to those wanting to adapt evidence-based body image and mental health curricula for LMIC contexts.

### Strengths

A core strength of this project is the participatory approach adopted by the multi-stakeholder project team, which has led to careful, culturally-sensitive co-creation of the intervention and provides opportunity for widespread dissemination across the country. Embedded within UNICEF Indonesia’s LSE curriculum, *Dove Confident Me Indonesia: Single Session * is projected to reach hundreds of thousands of adolescents attending state-run junior high schools by the end of 2022. This meets calls for intervention research to centre implementation planning at the core of development and design from both within the field of body image [66] and mental health practice more broadly [47]. By embedding our intervention within an established, acceptable framework and dissemination modality (i.e., UNICEF Indonesia’s LSE) we are ensuring the programme is set up for widespread dissemination and implementation. Importantly, as the dissemination plan has already been arranged with project stakeholders, namely UNICEF and the Dove Self-Esteem Project, a due-diligence contingency plan to remove the body image lesson from the current version of UNICEF Indonesia’s LSE programme has been arranged should the results from this trial indicate the programme has unintended negative effects on adolescent well-being upon full analysis. However, given that this intervention is based on an existing evidence-based intervention and has been carefully co-created by international and local experts, teachers, and school children, we are confident it will have the hypothesised positive effects on adolescents’ well-being.

Relatedly, we have task-shifted the delivery of the intervention to community providers (i.e., teachers and school guidance counsellors). Again, this a crucial strategy in enhancing wide-scale dissemination and ensuring intervention sustainability in contrast to relying on highly trained specialist providers [67–69]. Additional strengths of this study design are the use of a cluster RCT design and the large sample size. The cluster RCT design helps circumvent contamination of the control group [52] and the targeted 2000 participants will ensure adequate power to this study, while accounting for potential participant drop-out at follow-up time points.

### Limitations

We acknowledge several potential study limitations. We transitioned this research and intervention delivery to an online modality in response to the ongoing COVID-19 pandemic and related restrictions. This means that our eligibility criteria are now limited to higher-income schools and participants than previously intended, as it is essential students and teachers are experienced in live online teaching via video-conferencing technology and that students have access to their own electronic device. This is not representative of all students attending state junior high schools across Indonesia. For example, according to internal monitoring reports at the start of the COVID-19 pandemic by the Basic Education Working Group [70], less than 20% of teachers surveyed reported using interactive video conferencing technology, such as Zoom or Google Meet, and only 25% of children living in urban areas have computers to use for home-based learning. While the government has since made efforts to provide data packages for students’ education, many students remain without access to digital devices. Nonetheless, an online delivery modality will circumvent ethical and safety risks associated with attempting to run a face-to-face intervention during the COVID-19 pandemic and will offer new insights into the delivery of online mental health interventions delivered by teachers more broadly.

Given Indonesia’s diversity, a related limitation is that results from the trial may not be generalisable to adolescents across Indonesia as we are only evaluating the lesson in a single city (i.e., Surabaya). However, preliminary field testing of the intervention topics and activities with teachers and adolescents was conducted in two provinces reflecting different majority ethnic groups and religion (West Papua and South Sulawesi) prior to finalising the intervention content, in addition to focus groups on topic content with adolescent girls in Greater Jakarta. Further, the Ministry of Education and Culture reviewed the intervention content prior to it being finalised for testing, and this review process included teachers, officials, and experts from across the country.

Finally, with regards to the study design, our study does not include an active control group. Among the recommendations provided by Schleider et al. for evaluating single-session evaluations is the use of a comparable control condition in order to be more confident about the specificity and magnitude of the intervention’s impact. Indeed, they state “any strong claims about SSIs [single-session interventions] require commensurately strong, relevant control conditions” ([53] p269). They suggest that such control groups “match the intervention as closely as possible on features that are not hypothesized to be active, therapeutic elements” ([53] p271). However, as this is the first evaluation of its kind in Indonesia, testing the effectiveness of the lesson in comparison to as a lesson-as-usual wait-list control is considered an important first step before the introduction of an active control, allowing us to test for absolute rather than relative effects [71]. Should we find positive results from this trial, a follow-up study including an active control will be an important future direction.

## Conclusion

In this protocol paper, we have presented a rationale for the development and evaluation for a mixed-gender, teacher-led, single-session body image intervention for use in Indonesian junior high schools for adolescents aged 12–15 years. We have detailed the collaborative development and theoretical underpinnings of *Dove Confident Me Indonesia: Single Session * as well as the eventual dissemination plan, made possible by project partners UNICEF and the Dove Self-Esteem Project. Based on a commitment to transparency, we have outlined the steps we plan to take to conduct the study to evaluate the effectiveness and acceptability of *Dove Confident Me Indonesia: Single Session,* which includes an initial pragmatic internal feasibility pilot. We deem the pilot study a crucial first step prior to conducting a fully powered RCT trial due to the need to adapt our research design warranted by the ongoing COVID-19 pandemic. Finally, we have provided a summary of the project’s strengths and limitations.

## Supplementary Information

**Additional file 1. **SPIRIT Checklist.

**Additional file 2:** Example Parent Information and Consent Form.

**Additional file 3:** Acceptability Focus Group Schedules.

**Additional file 4:** Debrief Sheet.

## Data Availability

Not applicable.

## Abbreviations

LSE: Life Skills Education
LMIC: Low- and Middle-Income Country
RCT: Randomised Controlled Trial
T1, T2, T3: Time one, Time two, Time three
BMI: Body Mass Index (kg/m^2^)
COVID-19: Contagious coronavirus disease caused by the SARS-CoV-2 virus

## Footnotes

1 U-Report is a free tool for community participation, designed to address issues the population cares about. https://ureport.in/about/

2 At time of writing, schools in Indonesia have been mostly been providing education remotely for a period of approximately 10 months and there is not a certain indication on when schools will resume to full-time face-to-face learning. Consequently, conducting the trial online was both the safest and most pragmatic option to avoid substantial project delays.

3 Girl Effect is an international non-profit organisation that builds media content with the aim of arming girls with skills to make positive choices and changes in their lives during adolescence.

4 Exact shades: 4R15, 4R14, 1R13, 1Y12, 1Y10, 1R08, 2Y06, 1R02, and 5Y01
