# Supplementary material for: ‘Dove Confident Me Indonesia: Single Session’: study protocol for a randomised controlled trial to evaluate a school-based body image intervention among Indonesian adolescents
Source: BMC Public Health. 2021 Nov 16;21:2102. doi: 10.1186/s12889-021-11770-0 (PMC8593637; doi:10.1186/s12889-021-11770-0)
Supplement: Supplementary file 2 — Additional file 2: Example Parent Information and Consent Form. [file 12889_2021_11770_MOESM2_ESM.pdf]

Dove Confident Me Indonesia | Information Form

Your child's school has been selected to take part in a prestigious new research project on body image and self-esteem among Indonesian adolescents. Before you decide whether you are happy for your child to take part, it is important for you to understand why the research is being conducted and what it will involve.

Please take time to read the following information carefully. If you have any questions, please contact your child's school or a member of the research team (details below).

Who is doing the research?

The international research team includes academics at three universities:

1. **The University of the West of England**
   - Dr Nadia Craddock PhD, EdM
   - Kirsty Garbett MSc
   - Zoë Haime MSc
   - Dr Heidi Williamson, Professor of Psychology
   - Dr Phillippa Diedrichs PhD, Professor of Psychology
2. **University of Hawaii**
   - Dr Ayu Saraswati PhD, Associate Professor of Gender Studies (Indonesian)
3. **University of Indonesia**
   - Dr dr. Bernie Medise SpA (K), MPH (Indonesian)
   - dr. Kholisah Nasution SpA (K) (Indonesian)

The research team also includes school guidance counsellor Chairunnisa Rizkiah M. Psi. T (Indonesian).

This project is part of a collaboration with **UNICEF Indonesia** and **the Dove Self-Esteem Project** (Unilever) – who are the project funders. This project has endorsement from the Indonesian **Ministry of Education and Culture** and approval from the Surabaya District Education Office. In addition, a team of local researchers (Cimigo) will work with your child's school to collect data for this research project.

What is the aim of the research?

Body image and self-esteem are important issues for adolescents in Indonesia. According to a recent U-Report (UNICEF, March 2020):

- Nearly 50% of school-age Indonesian girls and boys said they had been teased or bullied about their appearance.
- Approximately 50% have stopped themselves from doing things they would like to do because they were worried about how they look; and
- Almost 90% said they wanted to learn ways to improve how they feel about their body in school.

The aim of this research is to find out how to better support Indonesian adolescents feel confident about their appearance in school by testing a 90-minute lesson: *Dove Confident Me Indonesia*. We want to understand if young people like the lesson and if it improves their body confidence.

What will taking part in this research involve?

In total, around 12 junior high schools in Surabaya have been selected to take part in this important research. With the permission of your child's school, we are inviting all students from Grade 7 to 9 to take part in a 90-minute body image lesson called *Dove Confident Me Indonesia,* which will be delivered by a teacher at your child's school. The lesson is based upon an existing world-leading evidence-based body image programme, which has been shown to improve girls' and boys' body image in studies conducted in the UK, Australia, Portugal and India. The lesson addresses factors such as the media, social media and other influences that can have a negative influence on young people's body image and self-esteem.

In order to gather information on how we can further enhance this lesson, we will invite your child and their classmates to complete series of brief questionnaires three times over a period of ten weeks to gather their feedback on the lesson and to evaluate its impact on their body image and well-being. We will also ask a small group of students and their teachers to participate in short focus group discussions to talk about their experiences of taking part in the lesson in more detail. In total, involvement in this project will take approximately 3-4 hours of your child's time.

Due to the COVID-19 pandemic, we are currently conducting all aspects of this research online. Data packages will be provided for all participating students.

What are the possible benefits of taking part?

The advantage of taking this research is that your child learns very important things about media, friendship, communication, and other influences on body image and teen self-confidence. In addition, every student admits that they like learning that is interesting and fun. One study showed that engaging in a subject-like activity on body image had a positive effect on adolescent well-being. Each student who completes this research will be awarded a certificate for his / her valuable contribution from all research contributors. We will also provide incentives in the form of souvenirs for children.

What are the possible risks of taking part?

Any participation in research can raise sensitive issues, but also positive insights. It is entirely your child's choice as to what they want to share with the researchers. We will reassure your child that there are no right or wrong answers and that no judgements will be made on the basis of what they write. Similar questionnaires to the one that will be used in this study have been widely used by researchers at the Centre for Appearance Research, UWE Bristol with other children in this age group. To date, there have been no students which have exhibited distress after completing these measures.

We do not anticipate that taking part in the body image lesson or completing the surveys will cause your child distress. In the unlikely event that this occurs, support strategies will be put in place with your child's school. However, based upon our research to date with other young people, we expect the lesson will have a positive impact on them.

What about confidentiality?

The information your child gives us will be treated with the highest level of confidentiality. Your child will be assigned a unique participation code and their name and identity will never be connected to their responses. Information that would make it possible to identify your child or any other child will never be included in any sort of report. Their responses will be written up and the data may be published in an academic journal or elsewhere and although direct quotes from your child may be used in a paper or report, their name and identifying information will be kept anonymous. The data will only be accessible to those working on the project.

The lesson will be video recorded to help the local research team (Indonesia only) assess the quality of the lesson and how easy it was for the teacher to deliver the lesson. Once assessed by the Indonesian researchers, video recordings will be deleted. The focus groups will be video recorded by the local research team (Indonesia only) so the research team ensure all information shared is captured. These recordings will be transcribed and anonymised. International researchers will only receive copies of the anonymised transcripts. Once transcribed, recordings will be deleted.

Where will the results of the research study be published?

The results of our study will be analysed by the research team and used in a report to UNICEF, the Dove Self Esteem Project, your child's school and the Ministry of Education and Culture. The anonymised results will also be used in conference presentations and peer-reviewed academic papers, and may feature in podcasts and blogposts.

Who has ethically approved this research?

The project has been reviewed and approved by ethical approval from the University of the West of England (HAS.20.05.174) and the University of Indonesia (KET-1373/ UN2.F1/ETIK/PPM.00.02/ 2020).

What do I have to do next?

We would be very grateful if you provide consent for your child to take part in this study. Please complete the consent form sent to you by your child's teacher.

Contact for Further Information

If you would like further information about this study, or have any other questions, please contact members of the local research team:

[redacted]

Dr dr Bernie Medise, SpA(K),
Universitas Indonesia

Dove Confident Me Indonesia | Consent

Statement

I hereby declare that I have been given the opportunity to read the information provided and also to ask if I have any questions.

By completing this form, I confirm that:

- I am the parent/guardian of the child named below.
- I allow the children, named below, to take part in the *Dove Confident Me Indonesia* study.

Full name of parent/guardian:

Child's full name:

Child's school name:

Date:
