# Supplementary material for: ‘Dove Confident Me Indonesia: Single Session’: study protocol for a randomised controlled trial to evaluate a school-based body image intervention among Indonesian adolescents
Source: BMC Public Health. 2021 Nov 16;21:2102. doi: 10.1186/s12889-021-11770-0 (PMC8593637; doi:10.1186/s12889-021-11770-0)
Supplement: Supplementary file 3 — Additional file 3: Acceptability Focus Group Schedules. [file 12889_2021_11770_MOESM3_ESM.pdf]

Additional File 3. Acceptability Focus Group Schedules (Student + Teacher; English version)

UNICEF Indonesia RCT Trial | STUDENT Acceptability Focus Groups

---

PART 1. Evaluation of Dove Confident Me Indonesia – General feedback (25 minutes)

Positive:

- What did you like about the lesson?
  - *Prompt: Please explain and provide examples*
  - *Only if students are struggling to respond - Prompt: What do you think other adolescents your age might like about it?*
- Which activities did you like best?
  - *Prompt: Please explain and provide examples.*
  - *Only if students are struggling to respond - Prompt: What activities do you think other adolescents your age might like best?*

Negative:

- What did you dislike about the lesson?
  - *Prompt: Please explain and provide examples*
  - *Only if students are struggling to respond - Prompt: What do you think other adolescents your age might not like about it?*
- Were there specific activities of the lesson that you disliked or found boring?
  - *Prompt: Please explain and provide examples.*
  - *Only if students are struggling to respond - Prompt:* Which activities do you think other adolescents your age might not like or find boring?
- Was there anything you found hard or confusing about the lesson? If so, what?

Impact:

- Do you think the lesson has made a difference to you in any way?
  - *Prompt: Has the lesson changed anything about the way you think or what you do? At home? At school? With friends? When alone?*
  - *Prompt: Please explain and provide examples.*

PART 2. Evaluation of Dove Confident Me Indonesia - Appropriateness (15 minutes)

Gender:

- How relevant were the activities for girls? Ask all Give a score out of 10.
  - *Prompt: Please explain and provide examples.*
  - *Prompt: Are there ways we could make the lesson more suitable for boys?*
- How relevant were the activities for boys? Give a score out of 10.
  - *Prompt: Please explain and provide examples.*
  - *Prompt: Are there ways we could make the lesson more suitable for boys?*

Age:

- What grades do you think this lesson is suitable for?
  - *Prompt: Please explain.*

Ability:

- How relevant was the language in the student worksheets?
    - Prompt: Was there anything you felt particularly difficult to understand? E.g., terms.

PART 3: Evaluation of Dove Confident Me Indonesia – Comfort (10 minutes)

- How did you feel about discussing body image in front of others in your class?
    - *Prompt: How comfortable did you feel participating? In front of girls? In front of boys? Please explain and provide examples.*

- Was there anything that made you feel uncomfortable in the lesson?
    - *Prompt: Please explain and provide examples.*
- Do you think there's anything that might make others feel uncomfortable?
    - *Prompt: Please explain and provide examples.*

QUICK POLL SECTION: For each question, ask participants to raise their hands on Zoom. No discussion necessary unless specified.

Quick Poll 1: The programme was delivered on Zoom because of COVID. Did you have any technology-related challenges? (e.g., frozen screen, internet disconnected)

	**Yes**	**No**
**No. of students**		

Quick Poll 2: If you did have technology-related challenges, how bad were they?

	**Very bad – I had to give up**	**Bad – I could carry on, but it was difficult to follow what was happening**	**Fine – I could still carry on ok**
**No. of students**			

Quick Poll 3: Do you think adolescents in Indonesia should learn about body image in schools?

	**Yes**	**No**
**No. of students**		

If no, ask students to explain why.

Quick Poll 4: Have you ever had lessons about body image in school before this lesson?

	**Yes**	**No**	
**No. of students**			

If yes, please can you tell us about the lesson.

Quick Poll 5: Have you ever learned about body image before this lesson outside of school?

	Yes	No
**No. of students**		

If yes, when? And what did you learn?

PART 4. Recommendations for future (10 minutes)

- If you could make any changes to the lesson for future students, what would they be? For example, this could be about any of the following -
    - Format (more/less group discussion, more/fewer writing tasks, a totally different method of learning?)
    - Length of session (longer/shorter/a series of lessons about body image so they can learn in more detail about the things discussed)
    - Content of sessions (was there an area of body image they felt was missing from the lesson? What was it?)

- Was there anything we haven't asked about regarding the lesson, that you would like to tell us?
- Do you have any questions for us?

Additional File 3. Acceptability Focus Group Schedules (Student + Teacher; English version)

UNICEF Indonesia RCT Trial | TEACHER Acceptability Focus Group

---

PART 1: Background and scene setting

1. Relevance of body image issues for Indonesian adolescents

- Do you feel body image concerns are relevant at your school?
    - *Prompt: Why or why not?*
- What age group did you deliver the lesson to?
- Do you think body image issues are relevant to this age group?
    - *Prompt: Please explain.*
    - *Prompt: Is there another age group that you think this lesson would be more appropriate for?*
- How important do you feel it is for students at junior high school to learn about body image?
    - *Prompt: Please explain.*

2. Prior teaching of body image

- Have you had experience delivering lessons in this informal student-led style before?
    - *Prompt: If yes, please explain.*
- Have you any previous experience of teaching similar topics before?
    - *Prompt: For example, lessons around puberty, self-esteem, well-being?*
    - *Prompt: If yes, please explain these lessons, and whether they were lesson plans made by yourself, the school, or accessed from elsewhere.* How did these lessons differ from Dove Confident Me Indonesia?

3. Teaching on Zoom

- How do you find teaching online?
    - *Prompt: What do you like / dislike?*
- Do you think this lesson would be received differently by students in a face-to-face setting?
    - Prompt: *If yes, please explain.*
    - Prompt: *In terms of student engagement? Behaviour management? Anything else?*

PART 2: Specific lesson feedback

2. Overall impressions of the lesson

- Did you enjoy teaching this lesson?
    - *Prompt: Why? Why not?*
- Did you think students enjoyed this lesson?
    - *Prompt:* Why/why not? Any aspects in particular that you think they enjoyed/didn't enjoy?

- Do you think there was anything in the lesson that students found hard to understand?
    - *Prompt: if yes, please g*ive examples.

- Do you think the lesson content was new and/or interesting for students?
    - *Prompt: if yes,* please explain, giving examples where possible.
- How did you find the timing/pace of the activities?
- Do you think this lesson is relevant for Junior Secondary Schools across Indonesia?
    - *Prompt:* Why / why not?
- Would you modify any of the language in order to aid students understanding?
    - *Prompt: if yes, c*an you provide any examples of this?
- Are there any key concepts you would like to see covered that aren't already?
    - *Prompt: if yes, please explain*

3. Teacher guide feedback

- What did you think of the Teacher Guide?
    - *Prompt: What did you like/not like?*
    - *Prompt: Did you find it easy to follow?*
    - *Prompt: What did you think of the design?*
    - *Prompt: How was the language?*
- How can we improve the teacher guide for other teachers delivering this lesson?

4. Teacher training feedback

- What did you think of the teacher training?
    - *Prompt: What did you like/not like?*
- How can we improve the teacher training for other teachers delivering this lesson?
- How do you think we can support teachers to feel confident delivering this lesson?

5. Final Recommendations

- Would you recommend these workshops be delivered with mixed-gender groups in the future or would it be better the groups be split into single-gender groups?
    - Prompt: Please explain.
- Do you think you will use this lesson in the future?
    - Prompt: Why/why not? What might be the barriers to utilising this lesson in the future at your school?
- Are there any other factors you feel we should know about or consider before we offer the lesson to other schools in Indonesia?
- Do you have any other recommendations you would like to add?
- Is there anything else you would like to add, that you think would be important for us to know, that has not already come up in our discussion today?
