# Supplementary material for: ‘Dove Confident Me Indonesia: Single Session’: study protocol for a randomised controlled trial to evaluate a school-based body image intervention among Indonesian adolescents
Source: BMC Public Health. 2021 Nov 16;21:2102. doi: 10.1186/s12889-021-11770-0 (PMC8593637; doi:10.1186/s12889-021-11770-0)
Supplement: Supplementary file 4 — Additional file 4: Debrief Sheet. [file 12889_2021_11770_MOESM4_ESM.pdf]

Thank you for taking part in our research

You have helped create a body image lesson that will improve the lives of *adolescents across Indonesia*!

Below we answer some commonly asked questions about the research project.

What was this study about?

Over the past two years a team of like-minded scientists and industry leaders have been developing a school lesson with the aim of improving body confidence among Indonesian adolescents. Research shows that many Indonesian adolescents feel unhappy about their appearance, and we wanted to develop positive content to change that. Pretty cool, huh?

YOU have been involved in a research project that evaluates how effective this lesson is. Has it worked? We hope so! We'll be examining all the responses we've had from our participants over the next couple of months.

How will this content be available to Indonesian adolescents?

The lesson will be available for state high schools across Indonesia as part of UNICEF's Life Skills Education Curriculum.

Where can I find out more?

You can find out more about the research being conducted on the Centre for Appearance Research website here: https://www.uwe.ac.uk/research/centres-and-groups/appearance

Help! I need more support with my body confidence and self-esteem.

We all need a little extra support sometimes. If you find yourself struggling with your well-being or self-esteem, here are some resources you might find useful.

SobatASK is an online counselling service, focused on sexual and reproductive health. It is available 24/7: https://sobatask.net/.

TePSA is a government run counselling service for all child protection concerns, including sexual abuse and depression or suicidal thoughts. It has nationwide availability, 24 hours a day, 7 days a week. The number to access the services is 021-1500771.
